# Supplementary figures and images for: Targeting tumor-intrinsic TAK1 triggers anti-tumor immunity and sensitizes pancreatic cancer to checkpoint blockade
Source: bioRxiv. 2025 Oct 9:2025.10.08.681226. Preprint. [Version 1] doi: 10.1101/2025.10.08.681226 (PMC12632323; doi:10.1101/2025.10.08.681226)

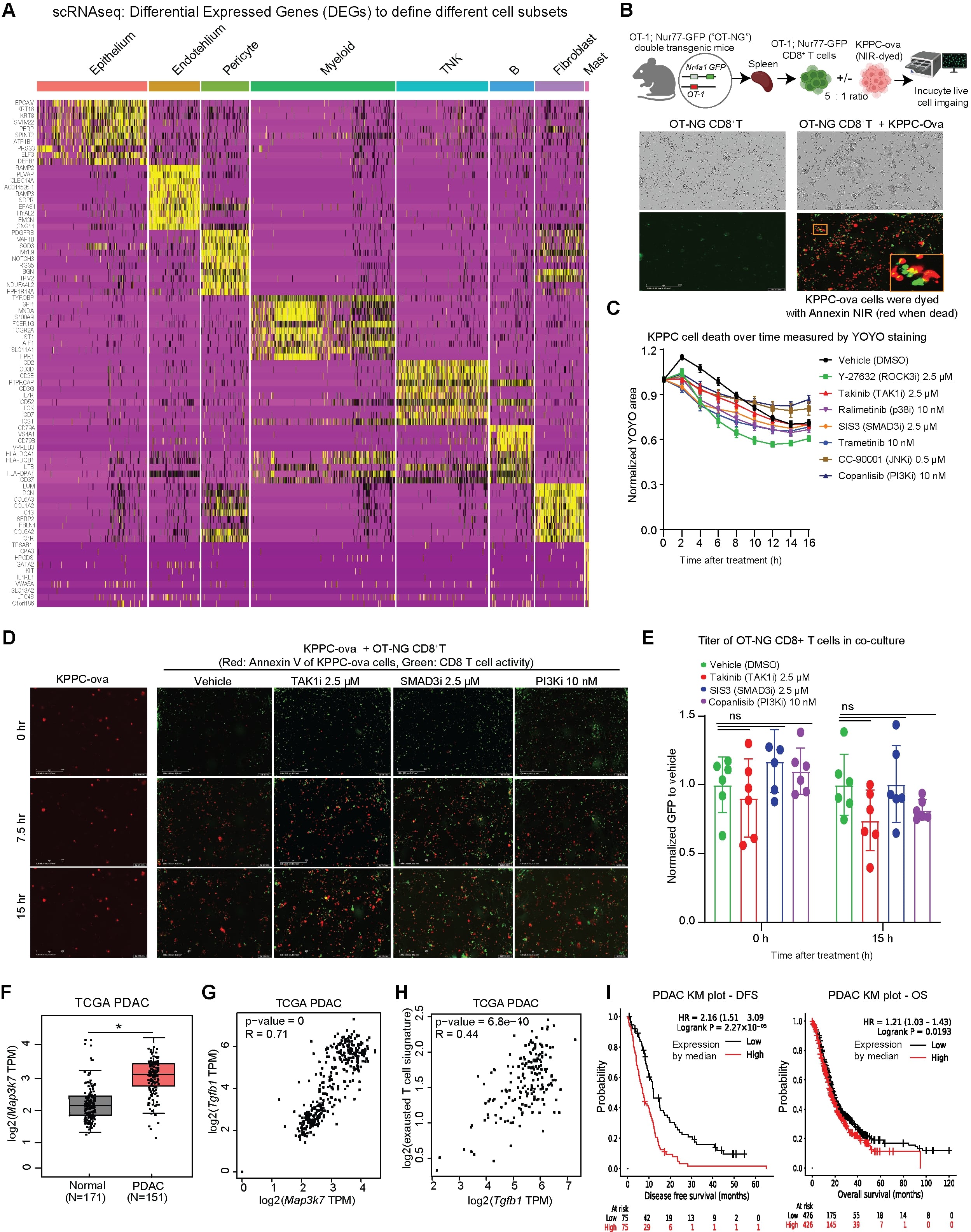

Supplement: Supplement 1 [file media-1.jpg]

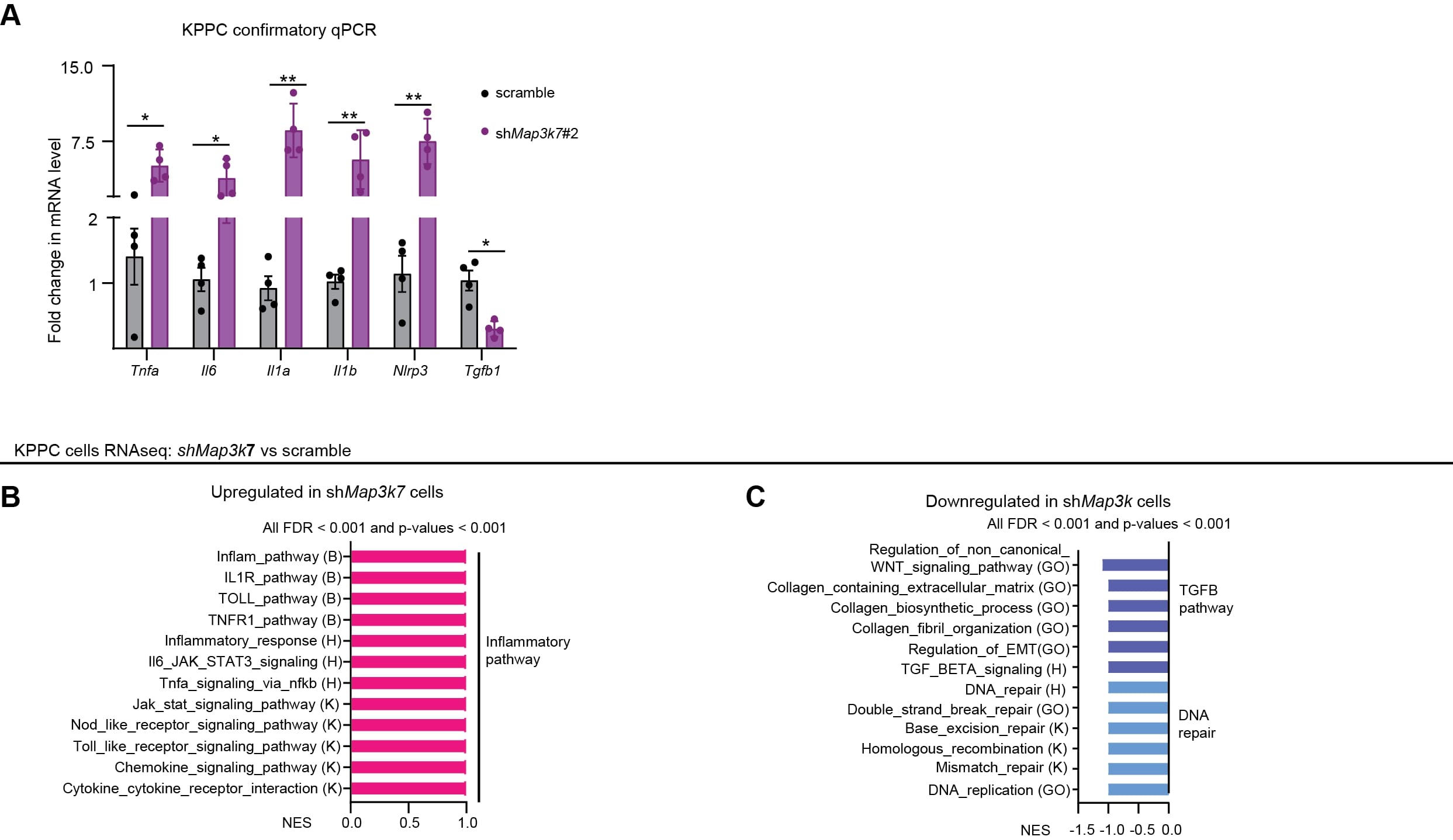

Supplement: Supplement 2 [file media-2.jpg]

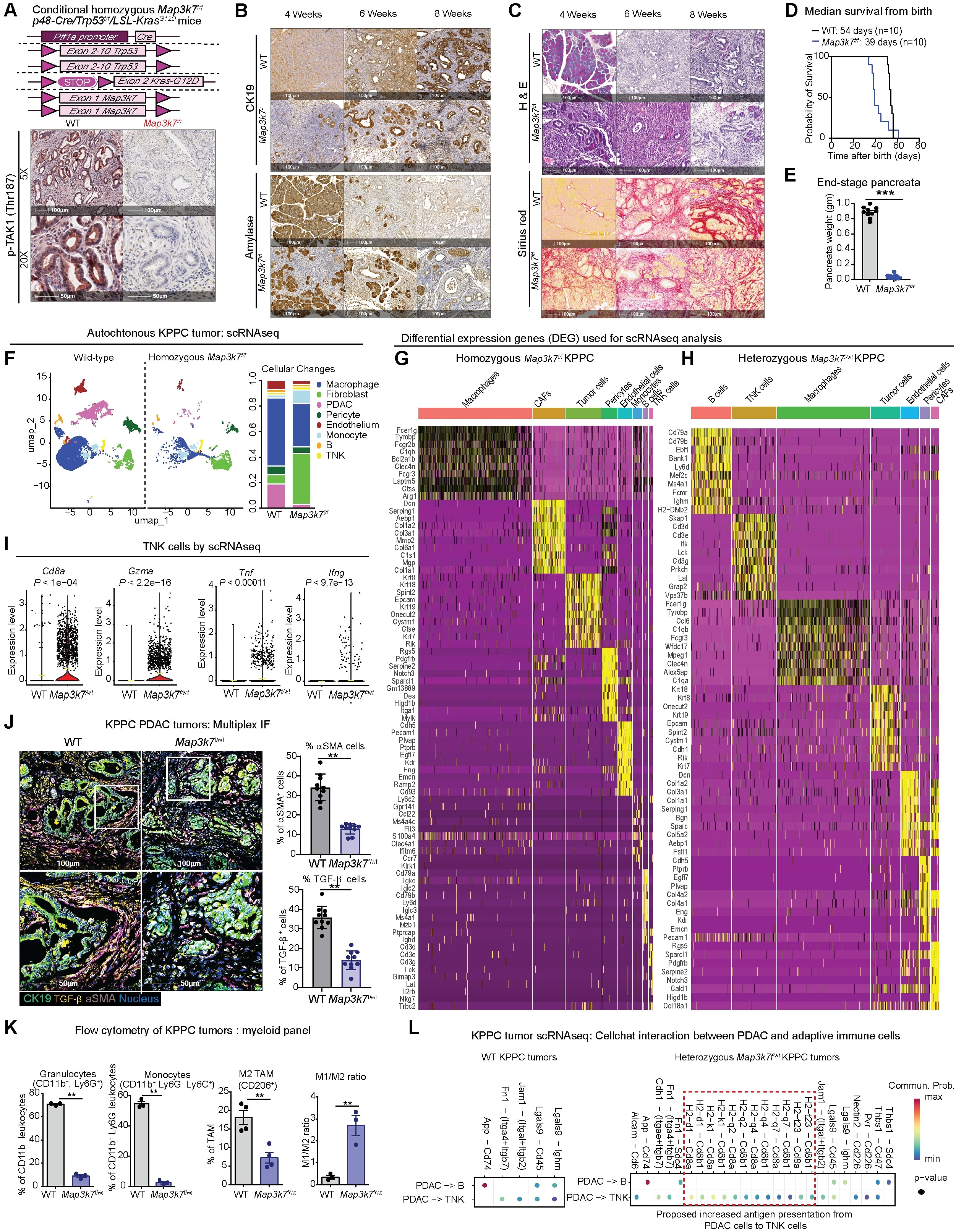

Supplement: Supplement 3 [file media-3.jpg]

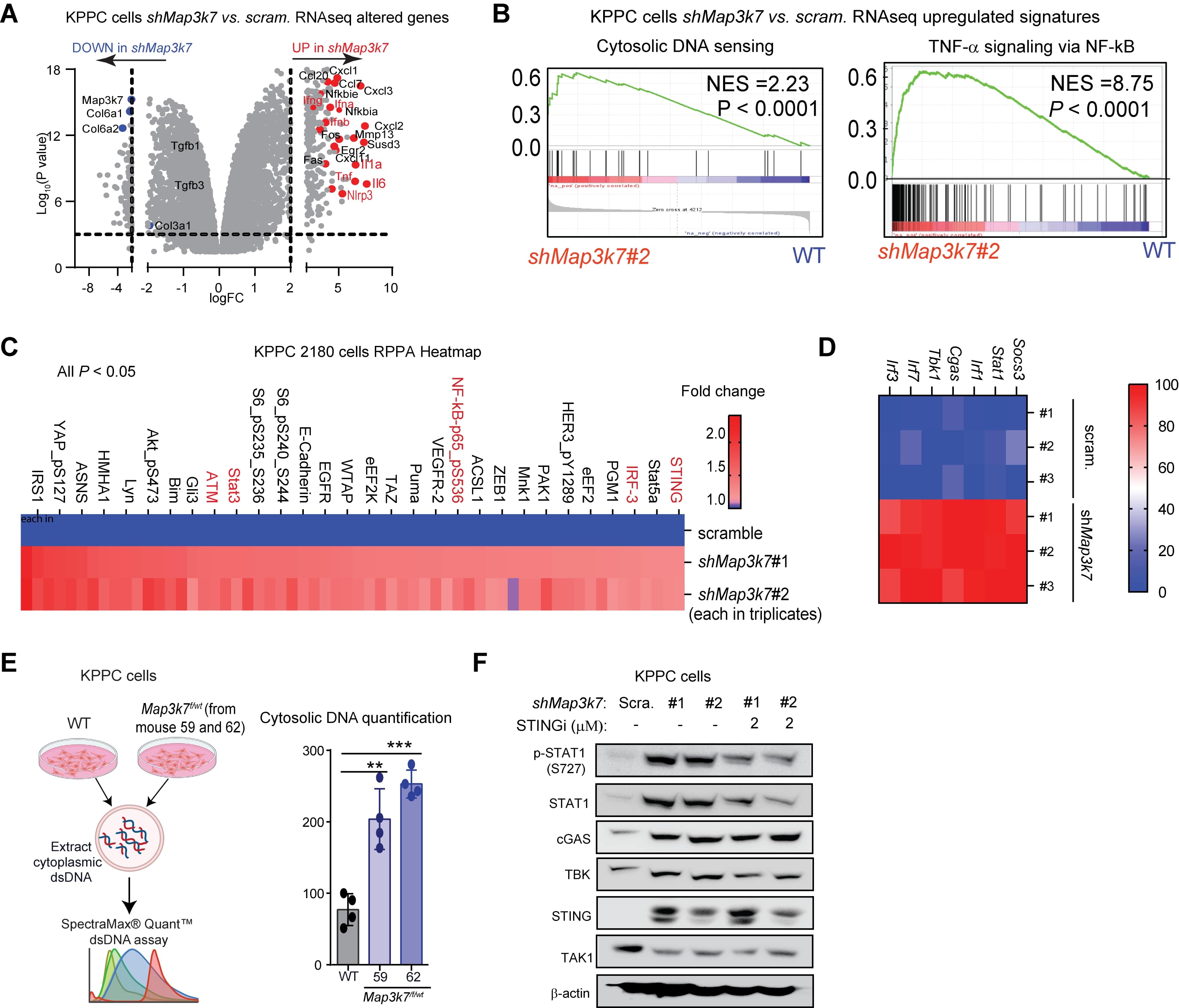

Supplement: Supplement 4 [file media-4.jpg]

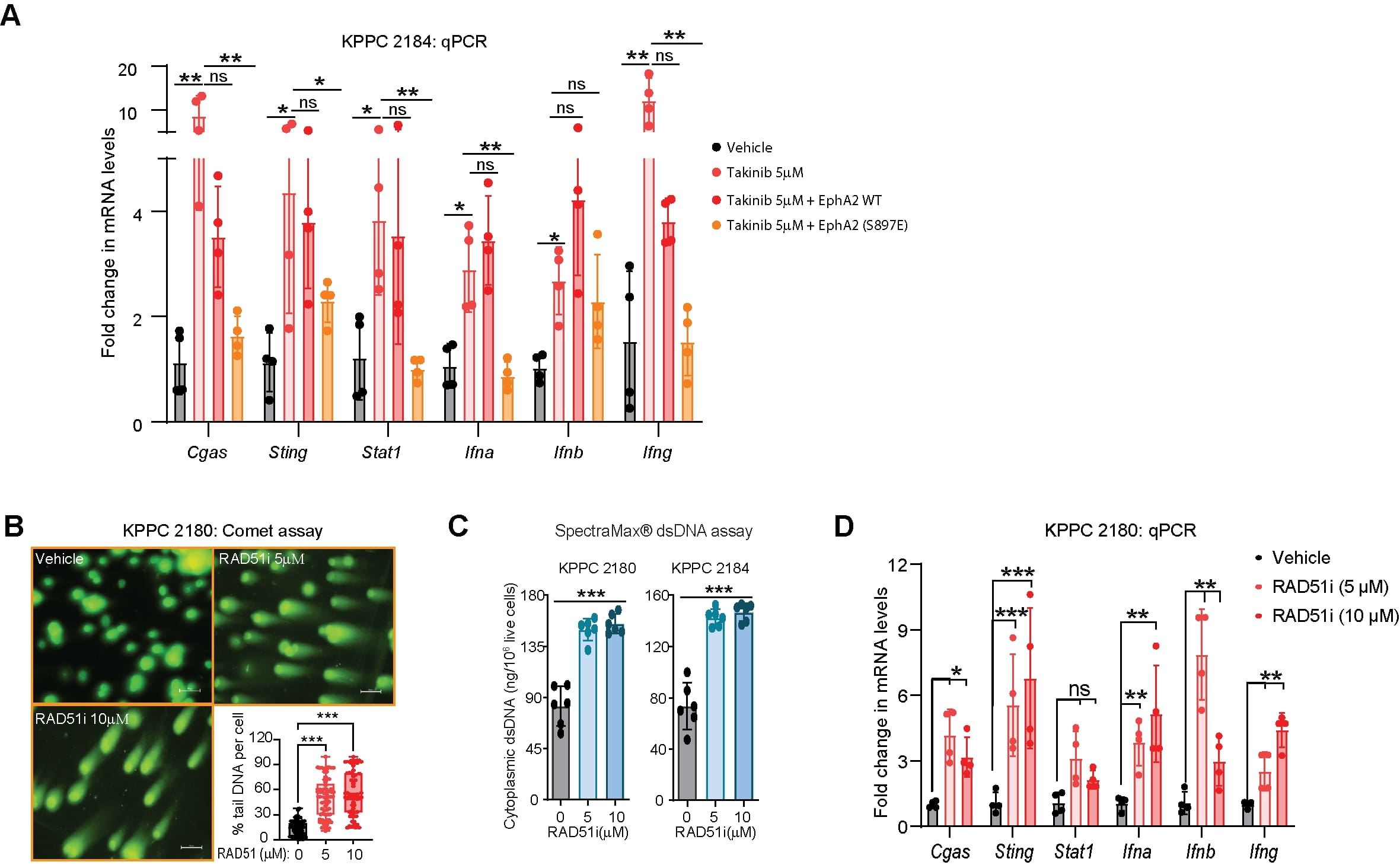

Supplement: Supplement 5 [file media-5.jpg]

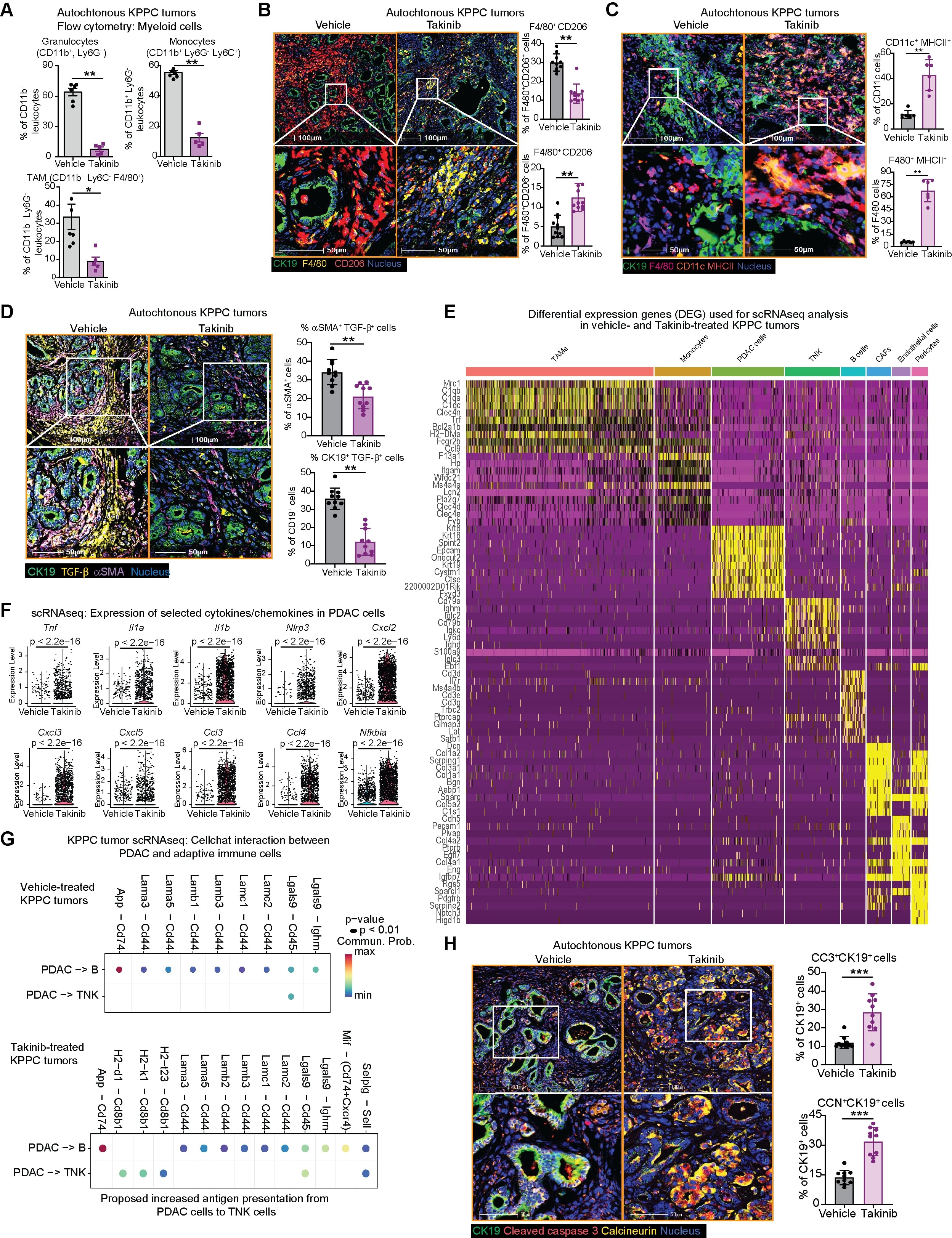

Supplement: Supplement 6 [file media-6.jpg]

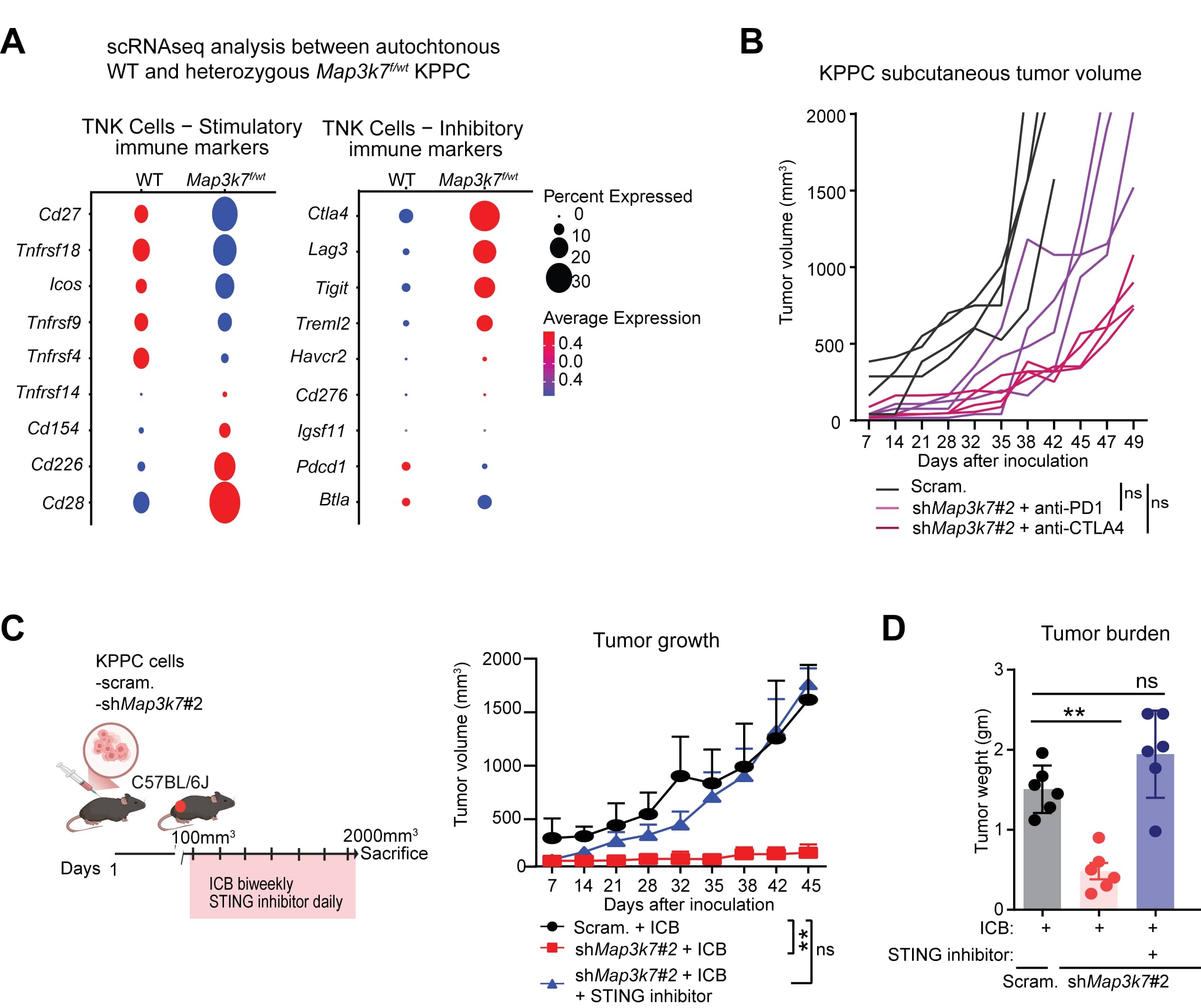

Supplement: Supplement 7 [file media-7.jpg]
